# On the Coordination Chemistry of the lanthanum(III) Nitrate Salt in EAN/MeOH Mixtures

**DOI:** 10.1021/acs.inorgchem.1c01375

**Published:** 2021-07-08

**Authors:** Valentina Migliorati, Alice Gibiino, Andrea Lapi, Matteo Busato, Paola D’Angelo

**Affiliations:** Dipartimento di Chimica, “La Sapienza” Università di Roma, P.le Aldo Moro 5, 00185 Rome, Italy

## Abstract

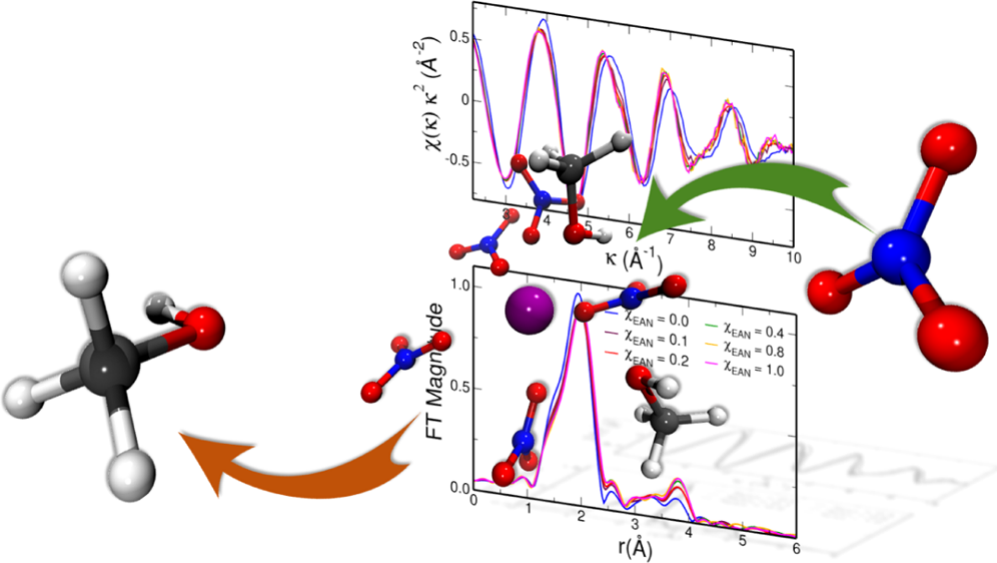

A thorough structural characterization of the La(NO_3_)_3_ salt dissolved into several mixtures of ethyl ammonium nitrate (EAN) and methanol (MeOH) with EAN molar fraction χ_EAN_ ranging from 0 to 1 has been carried out by combining molecular dynamics (MD) and X-ray absorption spectroscopy (XAS). The XAS and MD results show that changes take place in the La^3+^ first solvation shell when moving from pure MeOH to pure EAN. With increasing the ionic liquid content of the mixture, the La^3+^ first-shell complex progressively loses MeOH molecules to accommodate more and more nitrate anions. Except in pure EAN, the La^3+^ ion is always able to coordinate both MeOH and nitrate anions, with a ratio between the two ligands that changes continuously in the entire concentration range. When moving from pure MeOH to pure EAN, the La^3+^ first solvation shell passes from a 10-fold bicapped square antiprism geometry where all the nitrate anions act only as monodentate ligands to a 12-coordinated icosahedral structure in pure EAN where the nitrate anions bind the La^3+^ cation both in mono- and bidentate modes. The La^3+^ solvation structure formed in the MeOH/EAN mixtures shows a great adaptability to changes in the composition, allowing the system to reach the ideal compromise among all of the different interactions that take place into it.

## Abstract 2

The structural properties of the La(NO_3_)_3_ salt dissolved into EAN/methanol mixtures were characterized by molecular dynamics and X-ray absorption spectroscopy. The La^3+^ solvation shell undergoes significant changes with increasing the ionic liquid content of the mixture, progressively losing methanol molecules to accommodate more and more nitrate anions. The La^3+^ solvation structure shows great adaptability to composition changes, passing from a 10-fold bicapped square antiprism geometry in pure methanol to a 12-coordinated icosahedral complex in EAN.

## Introduction

Lanthanides and their derivatives have attracted much attention in the last years due to the emergence of novel application fields, including organic synthesis, catalysis, and medicine.^1−3^ In nuclear power technology and industrial processes, lanthanide 3+ (Ln^3+^) ions are separated by dissolving them into solvents with different polarities,^4^ and organic solvents have been widely used in the past to extract Ln^3+^ ions from the aqueous phase. In this respect, the possibility of employing ionic liquids (ILs) has been considered due to their several excellent properties and to their “green” characteristics.^5−10^ An accurate atomistic description of the Ln^3+^ ion solvation structures in IL media can thus be important also to select the best performing solvent. Moreover, the combination of lanthanides and ILs is important in many applications such as in ionothermal or microwave-assisted synthesis, for their use as luminescent hybrid materials and for metal electrodeposition.^11^ Among many investigations on mixtures of ILs and inorganic salts,^12−21^ several studies addressed the coordination chemistry of lanthanide ions in ILs showing a variety of solvation complex structures depending on the nature of the IL anions.^22−33^

**Figure 1:**
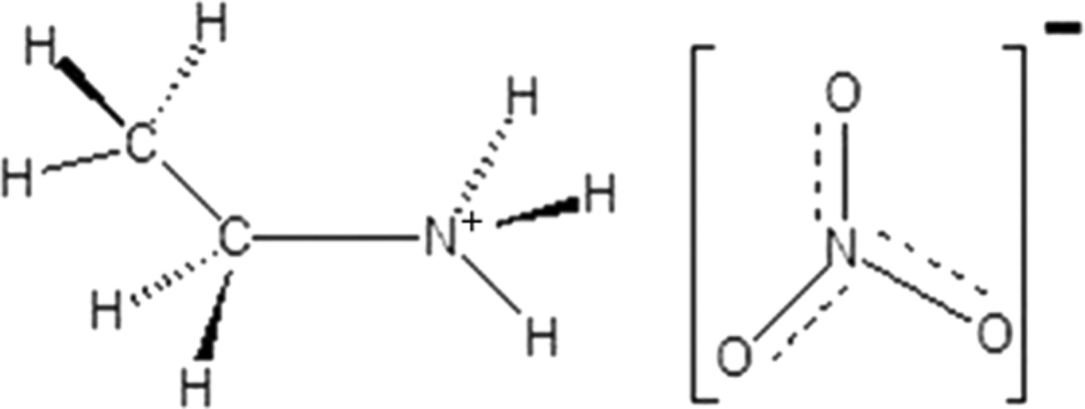
Structural formula of EAN.

It is well known that even if ILs possess many attractive and peculiar properties, they may present some obstacles when used in the pure state. For example, their high cost and high viscosity, together with the difficulty in obtaining them with high purity levels, limit their use in industry, at least on a large scale.^34−36^ Nonetheless, the physicochemical properties of ILs can be customized and optimized for instance by addition of cosolvents, such as water or alcohols, in order to obtain the desired characteristics for each purpose.^37−40^ Among the huge number of possible IL/cosolvent mixtures, the combination of ethyl ammonium nitrate (EAN) and methanol (MeOH) represents a very interesting system both from an academic and applicative point of view. EAN, whose structural formula is shown in Figure 1, is indeed one of the most widely investigated protic ionic liquid (PIL), which is prepared by a proton transfer reaction and it is thus characterized by proton-donor and proton-acceptor sites. A typical consequence is the presence in EAN of a strong three-dimensional hydrogen bond network similar to the tetrahedral one that can be found in bulk water.^41^ Similarly to EAN, MeOH is a molecular compound with strong ability as both a hydrogen bond donor and acceptor, but the presence of the methyl group also allows one to gain insights into the interactions of the studied medium with an organic cosolvent.^42,43^ Moreover, both MeOH and EAN are amphiphilic compounds, being characterized by hydrophobic and hydrophilic moieties. The intrinsic nature of EAN and MeOH results in a variety of different interactions which can take place in EAN/MeOH mixtures. Such interesting systems have been investigated both from an experimental and theoretical point of view.^44,45^ Russina et al. explored the mesoscopic morphology of EAN/MeOH mixtures using neutron and X-ray diffraction measurements and EPSR modeling technique, finding that though macroscopically homogeneous, the mixtures were highly heterogeneous at a mesoscopic level.^44^ On the other hand, the results of a Molecular Dynamics (MD) investigation have suggested for such systems, a homogeneous mixing process of added cosolvent molecules, which progressively accommodate themselves in the network of hydrogen bonds of the PIL, at variance with their behavior in aprotic ILs.^45^ In this framework, it is very interesting to investigate the solvation properties of lanthanide nitrate salts in EAN/MeOH mixtures and to predict the influence of the mixture composition on such properties, which represents a challenge for both science and industry.

In this paper, we used MD in combination with X-ray absorption spectroscopy (XAS) to investigate the La(NO_3_)_3_ salt dissolved into several mixtures of EAN and MeOH with the EAN molar fraction (χ_EAN_) ranging from 0 to 1. Among other approaches, the combined use of MD simulations and XAS has been shown to be a very powerful approach to provide a reliable description of the solvation structure of monoatomic ions in liquid samples.^46−51^ In our previous investigation on EAN solutions of La(NO_3_)_3_, we found that the La^3+^ ion in EAN forms an icosahedral nitrato complex with a 12-fold cation-oxygen coordination.^28^ Here, our MD/XAS joint procedure allowed us to shed light into the peculiar coordination complexes formed by the La^3+^ ions in EAN/MeOH mixtures and to unravel the changes of the La^3+^ solvation shell that take place when the mixture composition is varied from pure EAN to the pure MeOH.

## Methods

### MD Simulation Details

MD simulations of 0.1 M solutions of the La(NO_3_)_3_ salt in mixtures of EAN and MeOH were carried out at six different χ_EAN_ values ranging from 0 to 1. The composition and size of the simulated systems are reported in Table 1. As concerns the force field parameters, MeOH was described by the OPLS/AA force field.^52^ For the partial charges of the ethylammonium cation, we used the OPLS/AA charges for ammonium ions,^52^ while all of the other force field parameters for the EAN IL were taken from the Lopes and Padua force field.^53,54^ Lennard–Jones parameters for the La^3+^ cations were those developed by us in ref (55). Mixed Lennard–Jones parameters for the different atom types were obtained from the Lorentz–Berthelot combination rules, with the exception of those related to the La–O_NO_3_^–^_ (where O is the oxygen atom of the nitrate anion) interaction that were developed ad hoc to describe the La(NO_3_)_3_ salt dissolved in EAN solution.^28^

**Table 1:** Size and Composition of the Simulation Boxes Investigated in This Work

χ_EAN_^a^ =	0.0	0.1	0.2	0.4	0.8	1.0
nLa^3+^^b^	5	5	5	5	5	5
n_NO_3_^–^_c	15	120	199	321	499	573
n_MeOH_^d^	1227	945	736	459	121	-
n_EtNH_3_^+^_^e^	-	105	184	306	484	558
box edge (Å)	43.80	42.67	42.05	41.66	42.33	43.67

a EAN molar fraction of the mixture.

b Number of La^3+^ cations.

c Number of NO_3_^–^ anions.

d Number of MeOH molecules.

e Number of ethylammonium cations.

The simulations were performed with the GROMACS package^56^ and they were carried out at 300 K in the *NVT* ensemble using the Nosé-Hoover thermostat.^57,58^ In all cases, the initial configuration was constructed by randomly positioning the ions and the MeOH molecules in a very large cubic simulation box (with the PACKMOL package^59^) that was then compressed in the *NPT* ensemble. The box edge length to be used in the production phase was then determined by equilibrating the system in the NPT ensemble at 1 atm and 300 K for about 5 ns. After an equilibration run of 10 ns, the simulations were then carried out in the *NVT* ensemble at 300 K for 20 ns for all the systems with the exception of the EAN solution (χ_EAN_ = 1.0) which was simulated for 100 ns. Note that we used different simulation times for pure EAN and for the mixtures because the diffusion of the species in solution is usually increased when a co-solvent is added to the IL and, as a result, a reliable description of the structural and dynamic properties of the mixtures can be obtained also by using a shorter simulation time as compared to pure ILs. As an example in previous MD studies on IL/water mixtures and IL/acetonitrile solutions of a Lanthanum salt, by adopting a simulation time of 6 and 20 ns, respectively, an accurate characterization of the system properties was obtained.^9,60^ The timestep used in the simulations was of 1 fs. Long-range electrostatic interactions were computed with the particle mesh Ewald method,^61^ while a cut-off distance of 12 Å was adopted for the nonbonded interactions. The LINCS algorithm^62^ was employed to constrain the stretching interactions involving hydrogen atoms. The analyses of the MD trajectories were carried out using in-house written codes.

### X-ray Absorption Measurements

La(NO_3_)_3_·nH_2_O was purchased from Aldrich with a stated purity of 99.5%, and further purification was not carried out. The salt was then dried under Argon flux at 200° for 2 h to remove water, as previously reported.^63^ The 0.1 M solution of La(NO_3_)_3_ in MeOH was then prepared by dissolving the salt in anhydrous MeOH (Sigma-Aldrich). As concerns the 0.1 M solutions of La(NO_3_)_3_ in EAN/MeOH mixtures, the La(NO_3_)_3_ salt was first dissolved into an appropriate amount of EAN (Iolitec GmbH-stated purity of >99%) and kept under vacuum at 80 °C for 24 h to remove water,^28^ and then, an appropriate amount of anhydrous MeOH was added to achieve the desired molar ratio. All the operations were carried out under a vigorous dry Ar stream. The final water content determined by Karl-Fischer titration was lower than 150 ppm.

The La *K*-edge X-ray absorption spectra were collected at room temperature at the European Synchrotron Radiation Facility (ESRF), on the BM23 beam line, in transmission mode. The spectra were collected by using a Si(311) double-crystal monochromator with the second crystal detuned by 20% for harmonic rejection. For each solution, three spectra were collected and averaged. During the acquisition, the samples were kept in a cell with Kapton film windows and Teflon spacers of 2 cm. The XAS spectra were processed by subtracting the smooth pre-edge background fitted with a straight line by using the ATHENA code.^64^ The spectra were then normalized at unit absorption at 300 eV above each edge, where the EXAFS oscillations are small enough to be negligible. The EXAFS spectra have been extracted with a three segmented cubic spline and the corresponding Fourier transform (FT) were calculated on the *k*^2^-weighted χ(*k*)*k*^2^ function in the interval 2.2–10 Å^–1^ with no phase shift correction applied.

## Results

### XAS Data Comparison

**Figure 2:**
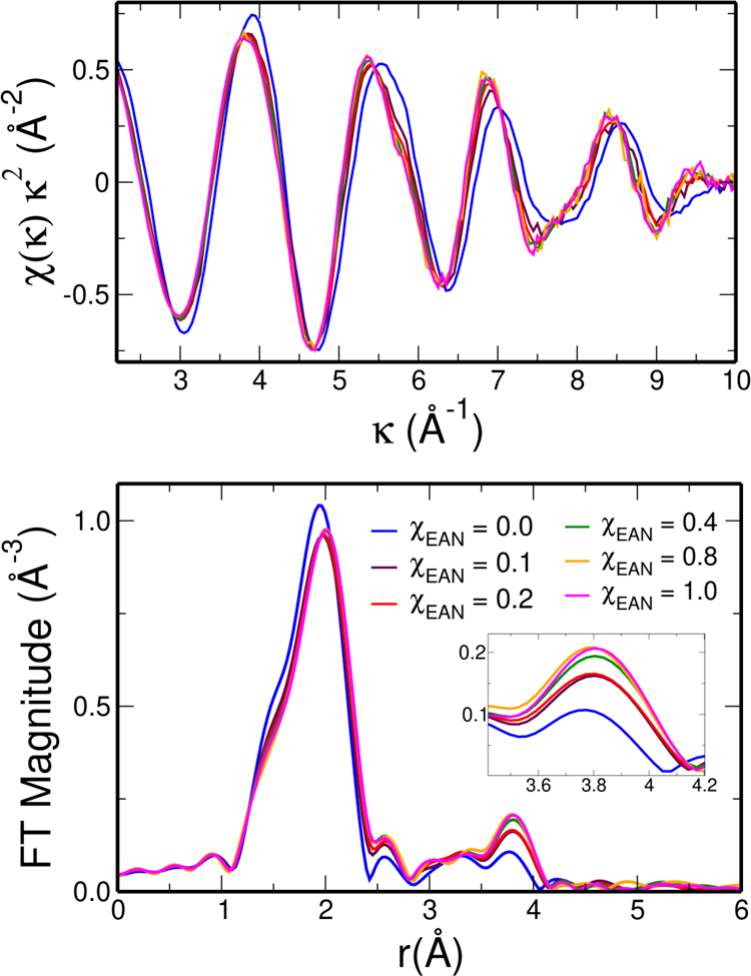
Upper panel: La *K*-edge experimental EXAFS spectra of the La(NO_3_)_3_ salt dissolved in EAN/MeOH mixtures with EAN molar fractions of 0.0 (blue lines), 0.1 (maroon lines), 0.2 (red lines), 0.4 (green lines), 0.8 (orange lines), and 1.0 (magenta lines). Lower panel: Non-phase shift corrected FTs of the experimental data reported in the upper panel.

Figure 2 shows the La *K*-edge EXAFS spectra of the La(NO_3_)_3_ salt in EAN/MeOH mixtures with χ_EAN_ = 0.1, 0.2, 0.4, and 0.8 together with the experimental data of the La(NO_3_)_3_ salt in pure MeOH and in the pure EAN. The EXAFS spectra show a main oscillation associated with the La–O first coordination shell whose frequency slightly increases with increasing EAN concentration. This behavior is due to the progressive appearance of a structural contribution at higher distances in the mixtures with a higher EAN content. This finding is confirmed by the trend of the FTs, as shown in the lower panel of Figure 2. Inspection of the figure shows that the FT first peaks of the EAN/MeOH mixtures and of pure EAN are almost superimposable, thus indicating that the La–O first coordination shell undergoes only small changes when the EAN concentration is decreased. Conversely, a more evident difference is observed when the La^3+^ cation is dissolved in pure MeOH, suggesting that a different coordination complex is formed in pure MeOH as compared to pure EAN. Moreover, a FT higher distance peak is found in the distance range between 3.5 and 4.0 Å whose intensity increases with increasing EAN concentrations. In a previous investigation on the solvation properties of Ce(NO_3_)_3_ in EAN, it was shown that such feature in the FT is due to multiple scattering effects of the nitrate ligands.^27^ Therefore, the increase of this peak intensity shows that at higher EAN concentrations, more and more nitrate anions enter the La^3+^ solvation complex. It is important to stress that very similar EXAFS spectra and FTs have been obtained for mixtures with similar compositions, such as those with χ_EAN_ = 0.1 and χ_EAN_ = 0.2, or with χ_EAN_ = 0.8 and χ_EAN_ = 1.0, suggesting that the structure of the La^3+^ ion first solvation shell is very similar in these composition ranges. Altogether, the EXAFS data provide a picture in which the La^3+^ first solvation shell in the EAN/MeOH mixtures progressively evolves from a coordination similar to that found in pure MeOH to the solvation shell formed in the pure EAN. During such transformation, it can accommodate more and more nitrate anions, probably at the expenses of other possible first-shell ligands, namely, the MeOH molecules.

### MD Results: Composition of the La^3+^ Ion Coordination Sphere

**Figure 3:**
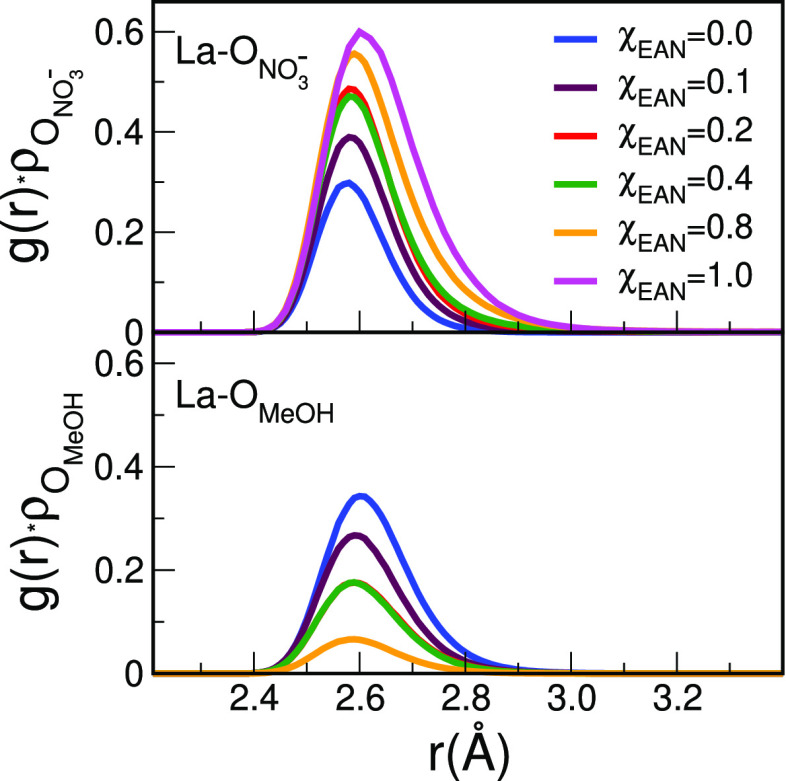
La–O_NO_3_^–^_ (upper panel) and La–O_MeOH_ (lower panel) radial distribution functions, where O is the oxygen atom of the nitrate anions and MeOH molecules, respectively, multiplied by the numerical density of the observed atoms, g(r)ρ′s, calculated from the MD simulations of the La(NO_3_)_3_ salt dissolved in EAN/MeOH mixtures with EAN molar fractions of 0.0 (blue lines), 0.1 (maroon lines), 0.2 (red lines), 0.4 (green lines), 0.8 (orange lines), and 1.0 (magenta lines).

To rationalize these results, we have carried out MD simulations of La(NO_3_)_3_ 0.1 M solutions in EAN/MeOH mixtures with χ_EAN_ = 0.0, 0.1, 0.2, 0.4, 0.8, and 1.0, that is, the same compositions used in the XAS experiments. The La–O_NO_3_^–^_ and La–O_MeOH_ radial distribution functions (*g*(*r*)’s) have been calculated from the MD trajectories, where O is the oxygen atom of the nitrate anions and of the MeOH molecules, respectively. Since when treating systems with different densities the simple comparison of the *g*(*r*)’s can be misleading,^65,66^ we show in Figure 3 the *g*(*r*)’s multiplied by the numerical density of the observed atoms (ρ). All of the *g*(*r*) structural parameters are listed in Table 2. Both the La–O_NO_3_^–^_ and La–O_MeOH_*g*(*r*)ρ′s show relevant differences when the EAN concentration of the mixture is varied. This result unambiguously shows that the La^3+^ first solvation shell undergoes changes when moving from pure MeOH to pure EAN, in agreement with the results obtained from the EXAFS experimental data. In particular, the intensity of the La–O_NO_3_^–^_*g*(*r*)ρ first peak significantly increases with the increasing EAN content, while the La–O_MeOH_*g*(*r*)ρ becomes less and less intense.

**Table 2:** Structural Properties Calculated from the MD Simulations of the La(NO_3_)_3_ Salt Dissolved in EAN/MeOH Mixtures^a^

χ_EAN_ =	0.0	0.1	0.2	0.4	0.8	1.0
La–O_MeOH_						
*R*_max_	2.59	2.59	2.59	2.55	2.58	
*N*_coord_	5.9	4.4	2.9	2.9	1.1	
La–O_NO_3_^–^_						
*R*_max_	2.58	2.59	2.58	2.59	2.59	2.59
*N*_coord_	4.1	5.8	7.5	7.7	10.0	11.7
La–O_MeOH_ + La–O_NO_3_^–^_						
*N*_coord_	10.0	10.2	10.4	10.6	11.1	11.7
La–N_NO_3_^–^_^(bi)^						
*R*_max_		3.16	3.15	3.14	3.12	3.11
*N*_coord_		0.1	0.3	0.6	1.8	3.6
La–N_NO_3_^–^_^(mono)^						
*R*_max_	3.73	3.73	3.73	3.73	3.73	3.74
*N*_coord_	4.1	5.6	6.9	6.5	6.4	4.5

a χ_EAN_ is the EAN molar fraction of the mixture. *R*_max_ is the position of the *g*(*r*) first peak, and *N*_coord_ is the corresponding average coordination numbers calculated for the La–O_MeOH_ and La–O_NO_3_^–^_ interactions. In both cases, the *N*_coord_ values were obtained by using a cut-off value of 3.30 Å. *R*_max_ and *N*_coord_ are reported also for the first (bi) and second (mono) peak of the La–N_NO_3_^–^_*g*(*r*)’s, where N is the nitrogen atom of nitrate anions. The cut-off values used to calculate *N*_coord_ for bidentate and mono-dentate coordination mode are 3.37 and 4.39 Å, respectively.

**Figure 4:**
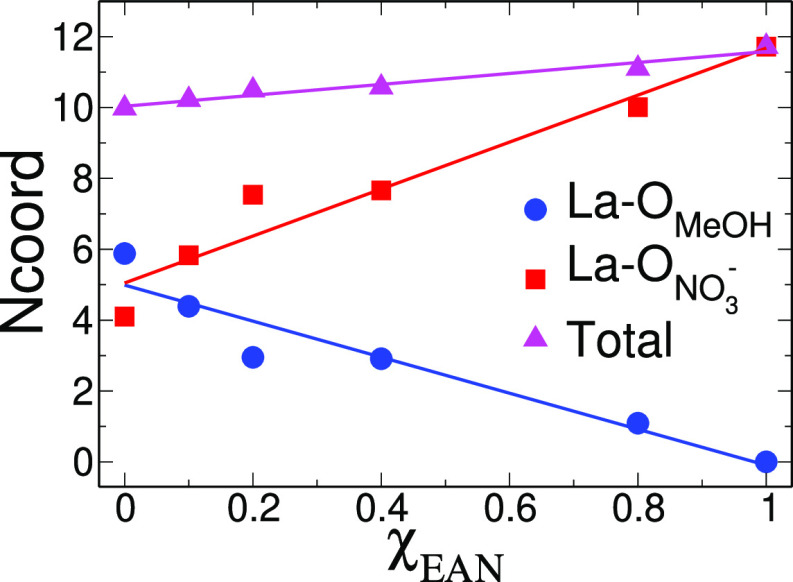
La–O_MeOH_ (blue circles), La–O_NO_3_^–^_ (red squares), and total La–O_MeOH_ + La-O_NO_3_^–^_ (magenta triangles) first-shell average coordination numbers (*N*_coord_) calculated from the MD simulations of the La(NO_3_)_3_ salt dissolved in EAN/MeOH mixtures as a function of the EAN molar fraction (χ_EAN_). Lines are inserted as a guide to the eye.

As concerns the distances, no significant variation of the position of the La–O_NO_3_^–^_ and La–O_MeOH_*g*(*r*) first peak is found when the composition is varied. Moreover, the La–O_NO_3_^–^_ first-shell distances tend to be similar to the La–O_MeOH_ ones. The La–O_NO_3_^–^_ and La–O_MeOH_ first-shell average coordination numbers (*N*_coord_) obtained by integration of the corresponding *g(r)* up to 3.30 Å are listed in Table 2. The variation of the obtained *N*_coord_ values has been reported also as a function of χ_EAN_ and is shown in Figure 4. The La–O_MeOH_*N*_coord_ significantly decreases with increasing the EAN content of the mixtures, while a simultaneous increase of the La–O_NO_3_^–^_*N*_coord_ is observed: going from low-to-high χ_EAN_, the La^3+^ first solvation shell progressively loses MeOH molecules to accommodate more and more nitrate ligands. Very interestingly, with the exception of the pure EAN system where no MeOH molecules are present, both the La–O_NO_3_^–^_ and La–O_MeOH_*N*_coord_ are always non-zero, indicating that the MeOH molecules and the nitrate anions are able to compete with each other to bind the La^3+^ metal ion in the entire concentration range. The La^3+^ first solvation shell is thus composed of both MeOH and nitrate ligands in pure MeOH and in all of the different mixtures. Altogether, these MD findings are in line with the progressive changes in the La^3+^ coordination environment that have been obtained from the trend of the EXAFS experimental data, in which the La^3+^ first solvation shell in the EAN/MeOH mixtures progressively evolves and at higher EAN concentrations, it accommodates more and more nitrate anions.

Note that the strong dependence of *N*_coord_ on the IL content of the mixture is lost when the total *N*_coord_ (La–O_NO_3_^–^_ + La–O_MeOH_) is calculated. By looking at the results shown in Figure 4, we can see that the total coordination number variation is of two units going from χ_EAN_ = 0.0 to χ_EAN_ = 1.0: the La^3+^ ion prefers to form a 10-coordinated first-shell complex in pure MeOH and a 12-fold structure in the pure EAN. The results obtained from the MD *g*(*r*)’s point to a very high affinity of the nitrate anions toward the La^3+^ cations which makes nitrate able to coordinate La^3+^ also when few nitrate anions are present in the system, namely, in the pure MeOH solvent. This is a very interesting result which is at variance with the behavior of La(NO_3_)_3_ in aqueous solutions, where the nitrate counter ion is not able to enter the La(NO_3_)_3_ ion coordination sphere as a consequence of the stronger solvation ability of water as compared to MeOH.^67^

**Figure 5:**
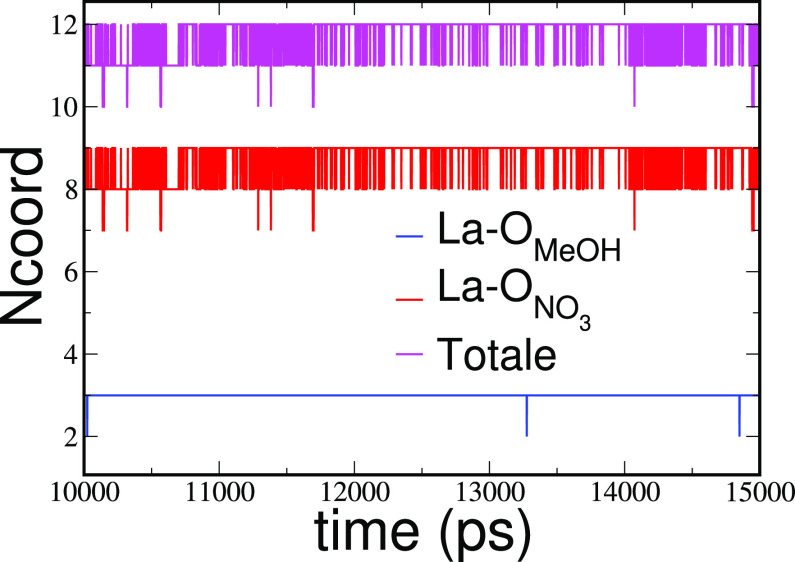
Time evolution of the La–O_MeOH_ (blue lines), La-O_NO_3_^–^_ (red lines), and total La–O_MeOH_ + La–O_NO_3_^–^_ (magenta lines) first-shell average coordination numbers (*N*_coord_) calculated from the MD simulations of the La(NO_3_)_3_ salt dissolved in the EAN/MeOH mixture with χ_EAN_ = 0.4.

It is interesting to point out that the non-integer values of coordination numbers obtained for La-O_NO_3_^–^_, La–O_MeOH_, and the total La–O_NO_3_^–^_ + La–O_MeOH_ interactions originate from both a static disorder for which different La^3+^ cations in the solutions show different *N*_coord_ and a dynamical disorder with *N*_coord_ changing their value in the course of the simulations. To give an idea of the ligand dynamics, we show in Figure 5 the evolution of the La–O_NO_3_^–^_, La–O_MeOH_, and the total La–O_NO_3_^–^_ + La–O_MeOH_*N*_coord_ calculated from the MD simulation of the mixture with χ_EAN_ = 0.4 for a single La^3+^ ion taken as an example. In particular, the *N*_coord_ values are plotted as a function of time in a time window of 5 ns and it can be clearly seen that several “jumps” of the *N*_coord_ values take place during the simulation.

**Figure 6:**
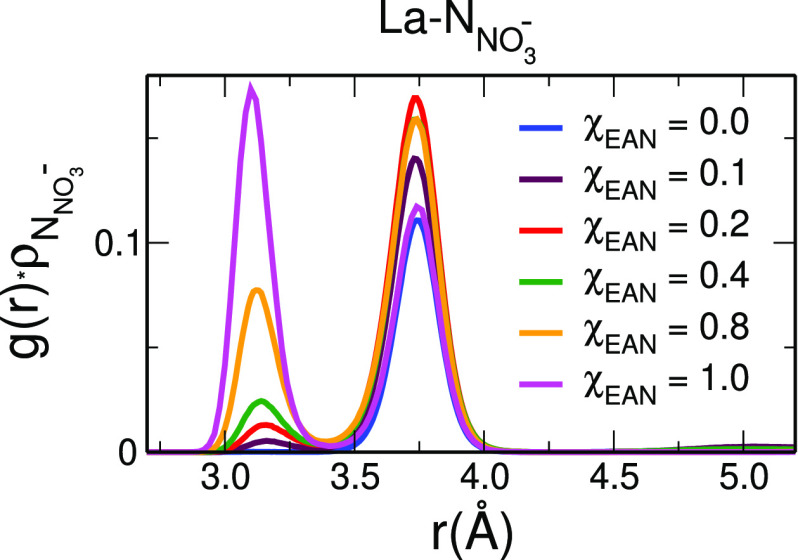
La–N_NO_3_^–^_ (N is the nitrogen atom of nitrate) radial distribution functions multiplied by the numerical density of the observed atoms, g(r)ρ′s, calculated from the MD simulations of the La(NO_3_)_3_ salt dissolved in EAN/MeOH mixtures with EAN molar fractions of 0.0 (blue lines), 0.1 (maroon lines), 0.2 (red lines), 0.4 (green lines), 0.8 (orange lines), and 1.0 (magenta lines).

It is well-known that the nitrate anions can act both as mono- and bidentate ligands when binding metal ions in liquid samples.^12,27,28,31−33^ It is therefore interesting to investigate the nitrate coordination mode toward the La^3+^ cations in the studied MeOH/EAN mixtures. Note that with bidentate coordination mode of nitrate, we refer to a chelating bidentate coordination, in which two oxygen atoms of a nitrate anion bind a single La^3+^ ion. To this end, we calculated from the MD simulations the *g*(*r*)’s between the La^3+^ ions and the N atom of the nitrate anions (La–N_NO_3_^–^_). The obtained *g*(*r*)ρ functions are shown in Figure 6, while the *g*(*r*) structural parameters are listed in Table 2.

As a first general trend, we observe remarkable differences in the La-N_NO_3_^–^_*g*(*r*)ρ′s when the EAN concentration of the mixture is varied. In pure MeOH, a single peak is observed, pointing to the existence of a single coordination mode, namely, the monodentate one. Conversely, two peaks are found in all of the other systems: a peak at low distances which is due to bidentate nitrate ligands and a second peak at higher distances due to nitrate anions that bind La^3+^ in a monodentate fashion. We can therefore conclude that when MeOH molecules are the main constituents of the La^3+^ first-shell complex, namely, in pure MeOH, the nitrate anions enter the shell by forming a monodentate coordination with La^3+^. On the contrary, when the La^3+^ first solvation sphere is mainly composed of nitrate anions, the nitrate anions can act either as a monodentate or a bidentate ligand. Overall, by looking at the La–N_NO_3_^–^_*N*_coord_ obtained by integration of the *g*(*r*)’s and listed in Table 2, it is evident that the nitrate anions prefer to bind the La^3+^ cation in a monodentate mode in all of the investigated systems, with a small percentage of bidentate coordination which becomes more and more significant with the increasing EAN content of the mixture and nitrate content in the La^3+^ first solvation complex. It is also interesting to understand if nitrate anions, besides acting as chelating bidentate ligands, are also able to form a bridge between two different La^3+^ cations. To this end, we analyzed the distribution of the nitrate-La^3+^ coordination numbers and our results showed that nitrate acts as a bridging bidentate ligand neither in the EAN/MeOH mixtures nor in the pure EAN solvent. Conversely, in the pure MeOH solution we found a significant percentage of clusters in which a single nitrate anion coordinates via two oxygen atoms two different La^3+^ cations (39%).

### MD Results: Structural Arrangement of the Nitrate Ligands Around La^3+^

**Figure 7:**
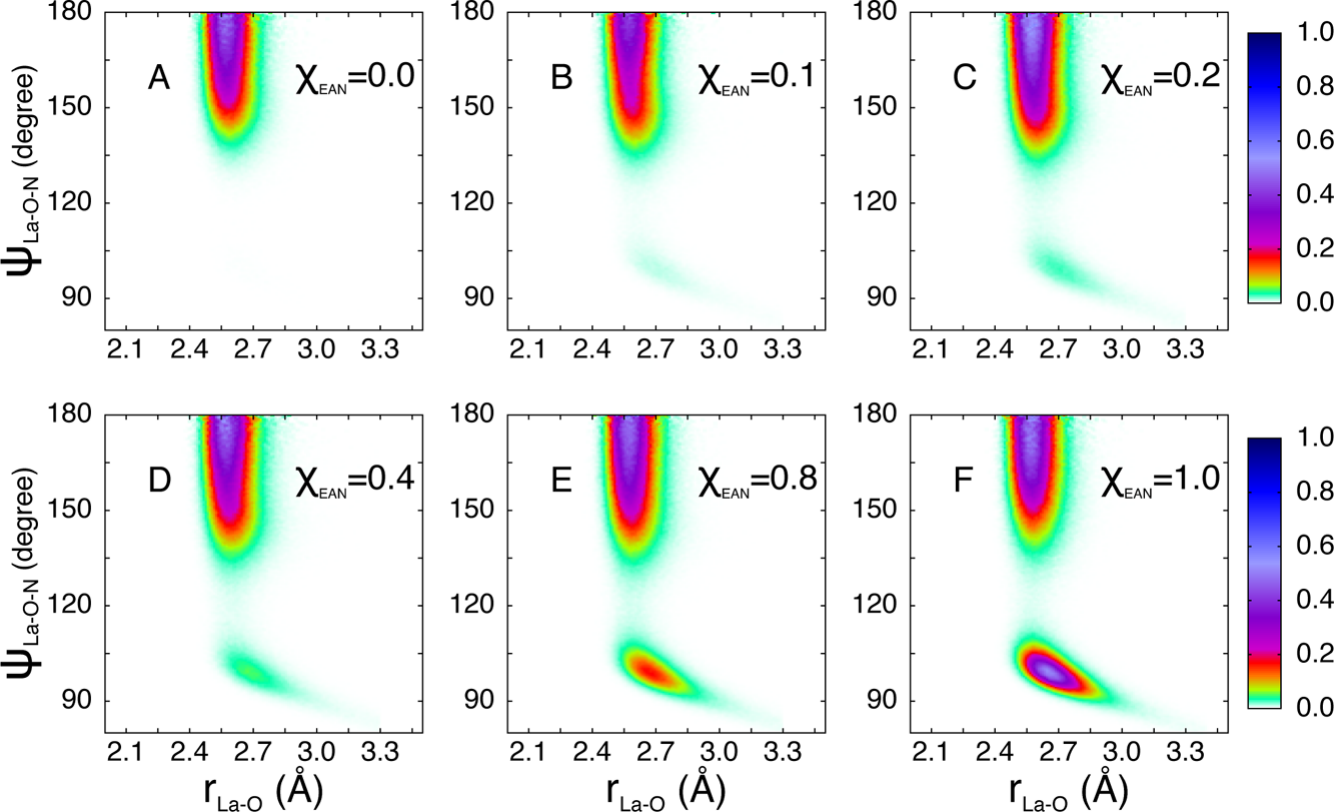
Combined distribution functions (CDFs) between the La–O distances and La–O–N angles where O and N are the oxygen and nitrogen atoms of the nitrate ion, respectively, calculated from the MD simulations of the La(NO_3_)_3_ salt dissolved in EAN/MeOH mixtures with EAN molar fractions of 0.0 (A), 0.1 (B), 0.2 (C), 0.4 (D), 0.8 (E), and 1.0 (F).

Additional insights into the geometric orientation of the nitrate ions in the La^3+^ first-shell complex can be gained by calculating from the MD simulations the CDFs between the La–O distances and the La–O–N angles, where O and N are the oxygen and nitrogen atoms of the nitrate ion, respectively (Figure 7). In these calculations, only the oxygen atoms belonging to the La^3+^ first coordination shell are considered (La–N_NO_3_^–^_ distance shorter than 3.30 Å) and only triplets of atoms La–O–N in which O and N belong to the same ligand. In the CDFs of all the investigated mixtures, a broad peak at angles between 150° and 180° is found, which is due to nitrate anions that coordinate the La^3+^ cation in a monodentate fashion. The broad shape of the peak indicates that the nitrate ions acting as monodentate ligands tend to align the O–N vector along the La–O direction forming a nearly linear La–O–N structure, but this configuration is very disordered and possesses a high orientational freedom. Conversely, the bidentate coordination gives rise to a peak centered at about 100° which increases in intensity as the EAN content of the mixture is increased. This peak is quite narrow as the simultaneous binding by La^3+^ of two oxygen atoms of a single nitrate ligand makes the resulting configuration rather fixed and less free to rotate.

**Figure 8:**
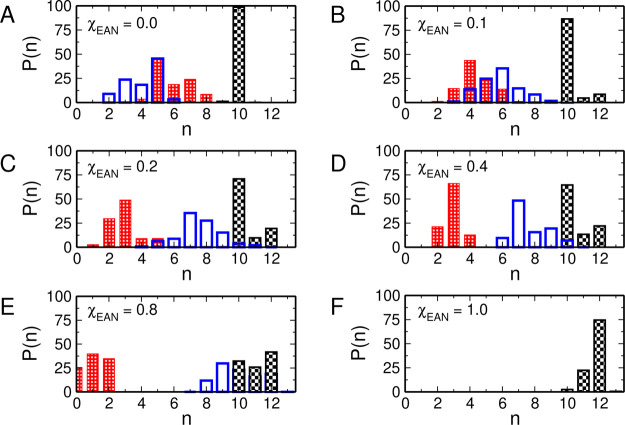
Coordination number distributions (*P*(*n*)) of the oxygen atoms of the MeOH molecules (red bars), of the oxygen atoms of the nitrate anions (blue bars), and of the sum of the oxygen atoms of the MeOH molecules and nitrate anions (black bars) in the La^3+^ first solvation shell calculated from the MD simulations of the La(NO_3_)_3_ salt dissolved in EAN/MeOH mixtures with EAN molar fractions of 0.0 (A), 0.1 (B), 0.2 (C), 0.4 (D), 0.8 (E), and 1.0 (F).

We have seen that at the higher EAN content of the mixture, the La^3+^ first solvation complex progressively loses MeOH molecules to accommodate more and more nitrate anions. A deeper insight into this behavior can be obtained by defining an instantaneous coordination number *n* of La^3+^ as the number of atoms of a certain type at a distance from La^3+^ shorter than 3.30 Å and analyzing its variation along the simulations. In particular, we have calculated the coordination number distributions for the O atoms of the MeOH molecules, for the O atoms of the nitrate anions and for their sum (Figure 8). As concerns the La–O_MeOH_ coordination number distribution, the most probable configurations gradually shift toward smaller coordination numbers when the MeOH concentration of the mixture is decreased. Note that in the mixture with χ_EAN_ = 0.8, the distribution shows significant percentages only of clusters with zero, one, or two MeOH molecules in the La^3+^ solvation complex. The La–O_NO_3_^–^_ coordination number distributions show the opposite trend, with the most probable configurations shifting toward higher values as the EAN content of the mixture is increased.

If one considers the La^3+^ first-shell complex constituted of either oxygen atoms of MeOH or of nitrate, a dominant percentage of the La^3+^ first coordination shell containing 10 first neighbors is found in pure MeOH and in all the mixtures with χ_EAN_ up to 0.4. Other possible configurations present in the mixtures are 11- and 12-fold clusters but, in all cases, the 10-fold complex is by far the most favorite one. Conversely, in the χ_EAN_ = 0.8 mixture and in pure EAN, the favorite complexes formed by La^3+^ ions are composed of 12 oxygen atoms of the first-shell ligands.

**Figure 9:**
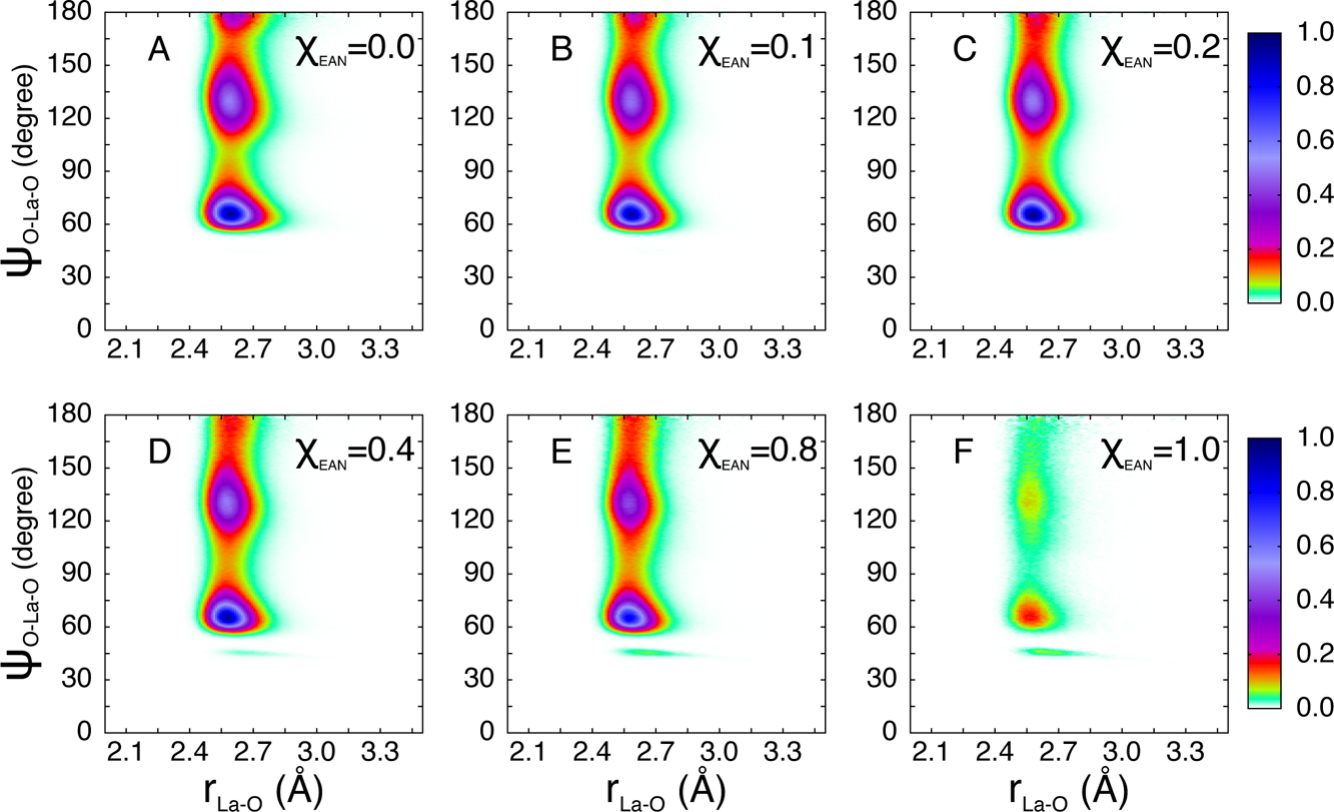
CDFs between the La-X distances and X-La-X angles evaluated for the 10-fold configurations only extracted from the MD simulations of the La(NO_3_)_3_ salt dissolved in EAN/MeOH mixtures with EAN molar fractions of 0.0 (A), 0.1 (B), 0.2 (C), 0.4 (D), 0.8 (E), and 1.0 (F). X is the oxygen atom of either MeOH molecules or of nitrate anions belonging to the first coordination shell of the La^3+^ ion.

In order to identify the global geometry of the coordination polyhedra formed by the La^3+^ ion in MeOH/EAN mixtures, we have resorted to calculate CDFs between the La–O distances and the O–La–O angles,^68^ where O is the oxygen atom of either MeOH molecules or of nitrate anions belonging to the first coordination shell of the La^3+^ ion. Note that this analysis has been carried out separately for the 10-fold, 11-fold, and 12-fold structures and by considering all of the oxygen atoms at a distance from the La^3+^ ion shorter than 3.30 Å. The CDFs calculated for the La^3+^ 10-coordinated first-shell clusters are shown in Figure 9. All of the CDFs show three peaks at about 65, 130, and 180°, pointing to the existence of a bicapped square antiprism geometry of the ten oxygen atoms surrounding the La^3+^ ion. Moreover, the distributions are similar in all the studied systems and this means that the 10-fold structure which is formed is able to accommodate both nitrate and MeOH ligands in a similar way, independently of the specific composition of the first-shell complex which undergoes strong modifications going from pure MeOH to the pure EAN system. Therefore, we can conclude that the bicapped square antiprism 10-fold complex has a great flexibility which allows the structure to accommodate different ligands without distorting itself in a significant way.

**Figure 10:**
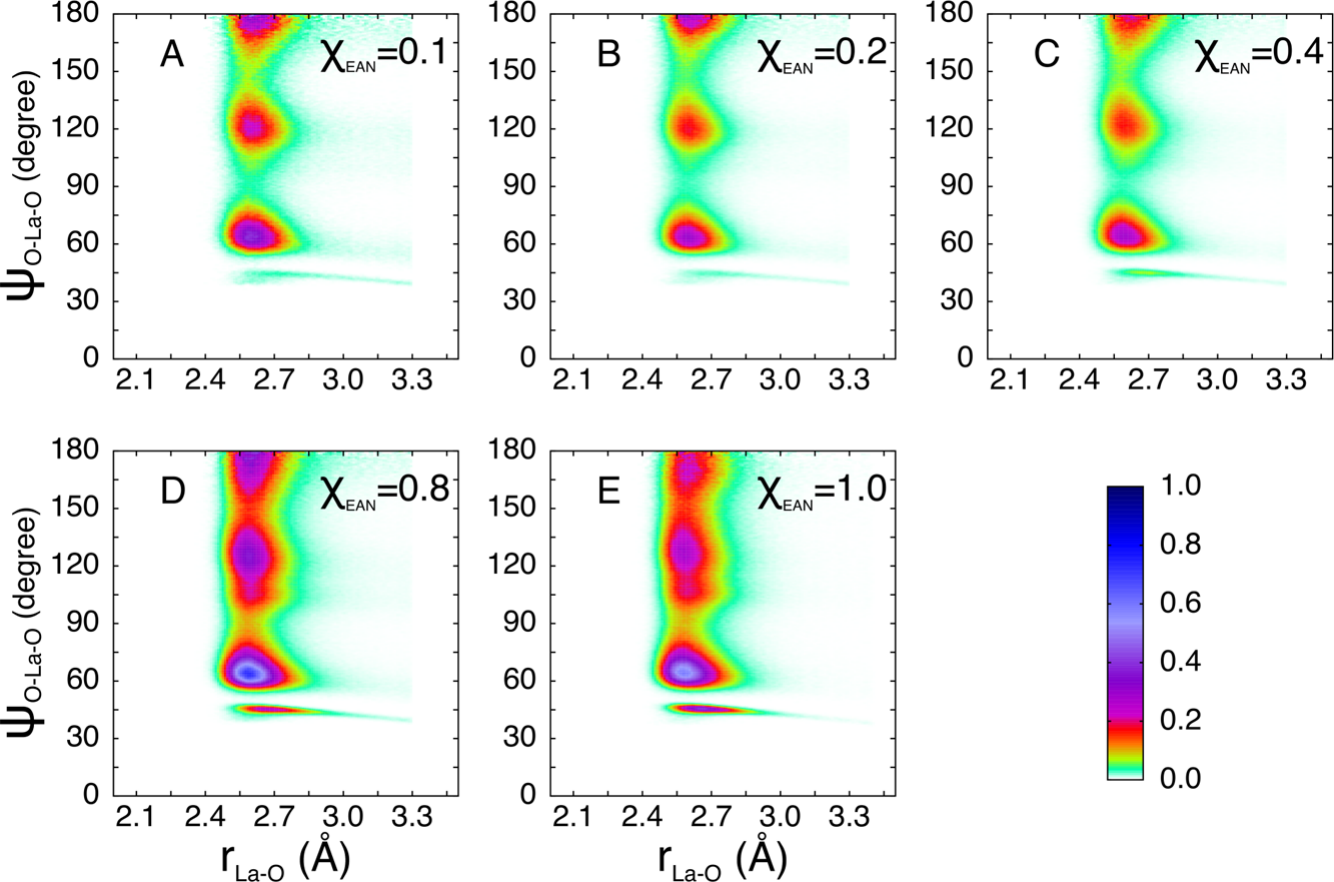
CDFs between the La–X distances and X–La–X angles evaluated for the 11-fold configurations only extracted from the MD simulations of the La(NO_3_)_3_ salt dissolved in EAN/MeOH mixtures with EAN molar fractions of 0.1 (A), 0.2 (B), 0.4 (C), 0.8 (D), and 1.0 (E). X is the oxygen atom of either MeOH molecules or of nitrate anions belonging to the first coordination shell of the La^3+^ ion.

The CDFs evaluated for the 11-fold complexes are reported in Figure 10 and show three well defined peaks at about 65, 120, and 180°. Note that these features are somewhat similar to the ones calculated for the 12-fold icosahedral geometry (vide infra), albeit significantly distorted. The distributions obtained are compatible with an edge-contracted icosahedral structure, a peculiar geometry in which two vertexes of a regular icosahedron are collapsed into one. Such structure could easily form around the La^3+^ cation when a first-shell oxygen atom of an icosahedral complex leaves, inducing a rearrangement of the remaining ligands.

**Figure 11:**
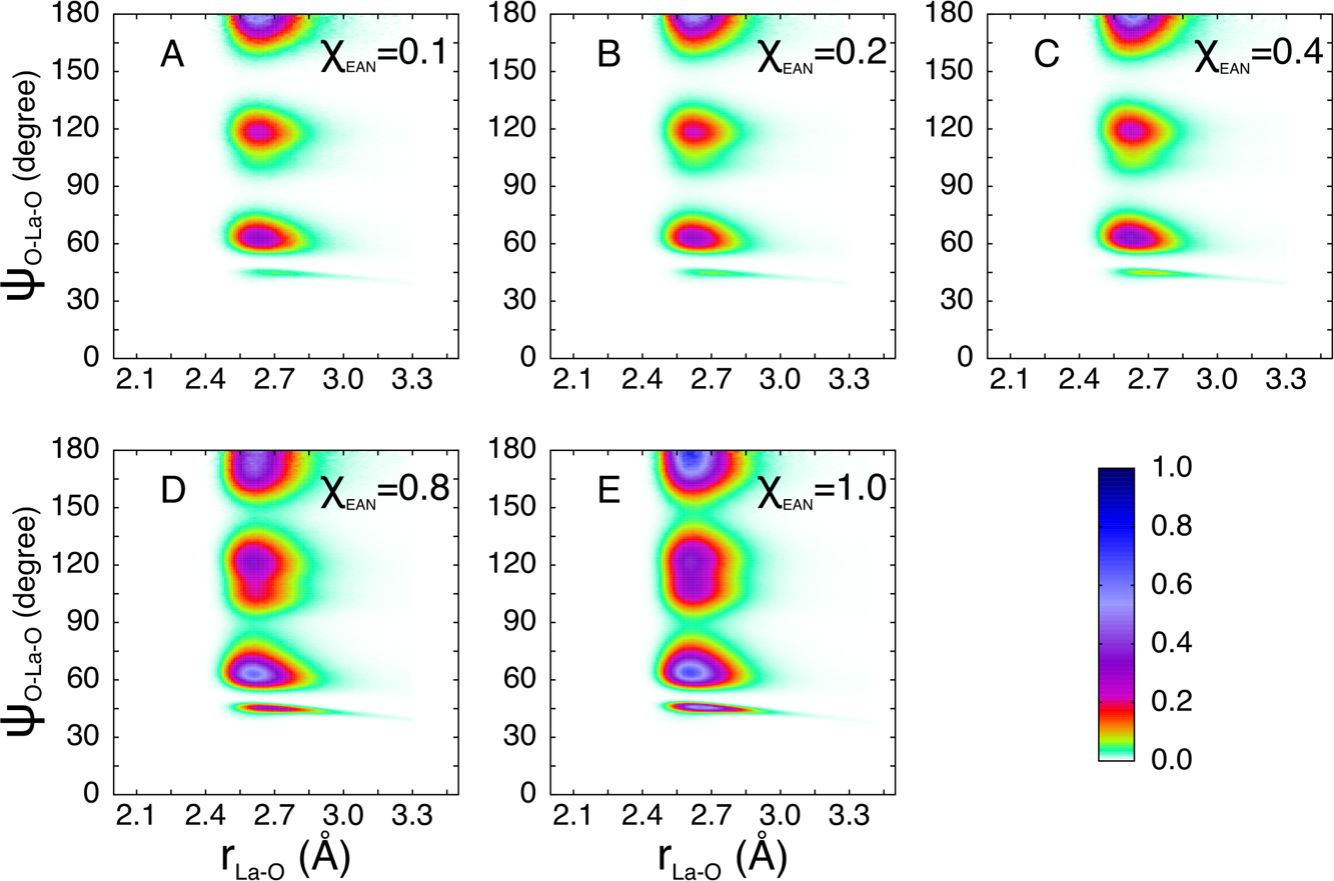
CDFs between the La–X distances and X–La–X angles evaluated for the 12-fold configurations only extracted from the MD simulations of the La(NO_3_)_3_ salt dissolved in EAN/MeOH mixtures with EAN molar fractions of 0.1 (A), 0.2 (B), 0.4 (C), 0.8 (D), and 1.0 (E). X is the oxygen atom of either MeOH molecules or of nitrate anions belonging to the first coordination shell of the La^3+^ ion.

**Figure 12:**
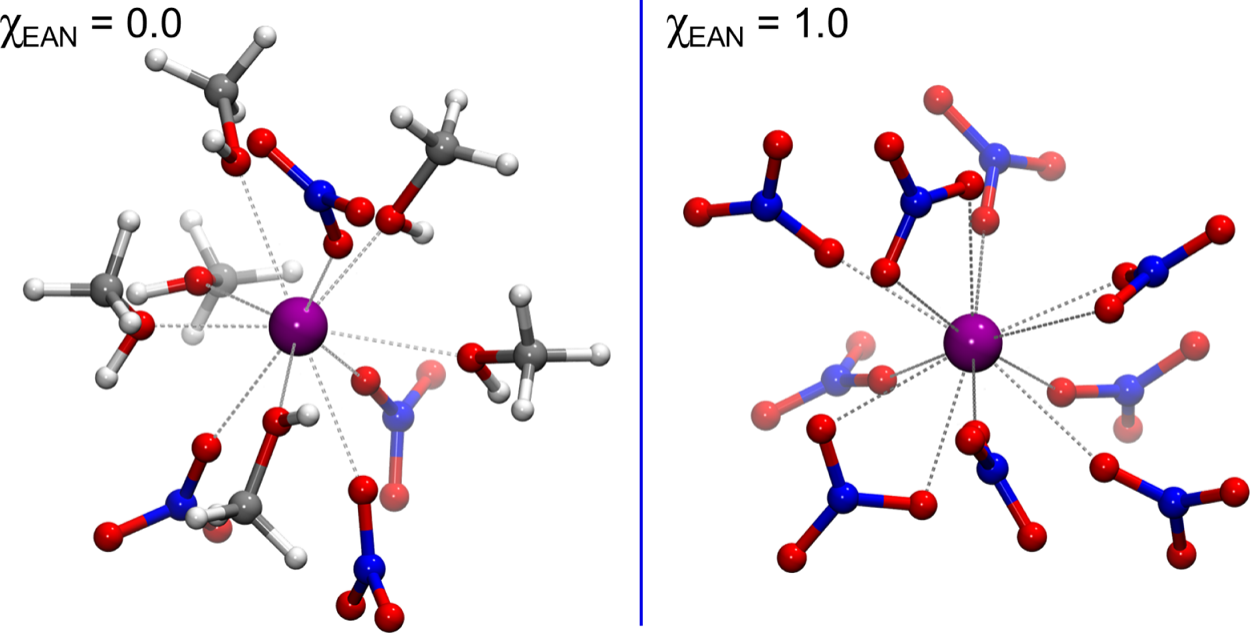
10-fold (left panel) and 12-fold (right panel) solvation complexes formed by the La^3+^ ion in pure MeOH and pure EAN, respectively, as obtained from two MD snapshots. Carbon, nitrogen, oxygen, hydrogen, and La atoms are represented in gray, blue, red, white, and purple, respectively.

As far as the 12-fold coordination is concerned (Figure 11), the CDFs show three peaks at about 65, 120, and 180°. The obtained distributions are compatible with the existence of an icosahedral geometry of the 12-fold La^3+^ first-shell complex. This result is in agreement with previous findings reported in the literature showing the existence of an icosahedral nitrato complex with a 12-fold coordination for the La^3+^ ion in EAN.^28^ The CDF peaks are well defined and separated in the mixtures with χ_EAN_ = 0.1, 0.2, and 0.4, indicating an ordered and regular first-shell geometry. Conversely, when the EAN content of the mixture is higher (χ_EAN_ = 0.8 and 1.0), broader peaks are found due to a more unstructured and distorted coordination structure. In Figure 12, two MD snapshots are shown highlighting, as examples, the coordination geometry of the La^3+^ solvation complexes in pure MeOH and pure EAN.

### MD Results: Analysis of Nitrate, Ethylammonium, and Methanol Interactions

**Figure 13:**
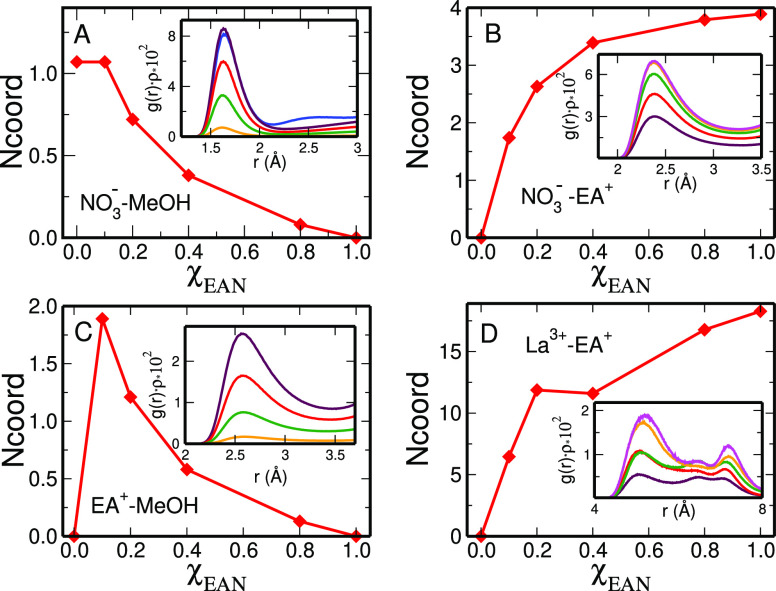
Panels: O_NO_3_^–^_–H_MeOH_ (A), O_NO_3_^–^_–H_EA^+^_ (B), H_EA^+^_–O_MeOH_ (C), and La^3+^–N_EA^+^_ (D) first-shell average coordination numbers (N_coord_) calculated from the MD simulations of the La(NO_3_)_3_ salt dissolved in EAN/MeOH mixtures as a function of the EAN molar fraction (χ_EAN_). Insets: O_NO_3_^–^_–H_MeOH_ (A), O_NO_3_^–^_–H_EA^+^_ (B), H_EA^+^_–O_MeOH_ (C), and La^3+^–N_EA^+^_ (D) radial distribution functions multiplied by the numerical density of the observed atoms, *g*(*r*)ρ′s, calculated from the MD simulations of the La(NO_3_)_3_ salt dissolved in EAN/MeOH mixtures with EAN molar fractions of 0.0 (blue lines), 0.1 (maroon lines), 0.2 (red lines), 0.4 (green lines), 0.8 (orange lines), and 1.0 (magenta lines).

Once the changes of the La^3+^ solvation structure have been assessed, it is very interesting to analyze how the interactions among the other species in the systems, namely, nitrate, ethylammonium, and MeOH are modified when the EAN content of the mixtures is varied. To this end, we have calculated from the MD trajectories the *g*(*r*)ρ functions of a selected subset of atoms, as shown in Figure 13, together with *N*_coord_ calculated by integration of the *g*(*r*)’s up to the first minimum. In pure MeOH, besides interacting with the La^3+^ ion, the nitrate anions are solvated by the MeOH molecules (panel A). In particular, on average, each oxygen atom of the nitrate anions (O_NO_3_^–^_) forms almost one hydrogen bond interaction with the hydrogen atom of the MeOH hydroxyl group (H_MeOH_). When the EAN IL is added to MeOH to form the mixture, nitrate anions form hydrogen bond interactions also with the cation of the IL, namely, ethylammonium (EA^+^), and the two kinds of interactions show, as expected, an opposite trend: the O_NO_3_^–^_–H_MeOH_*N*_coord_ decreases with increasing EAN concentration, while more and more interactions between the IL cations and anions are formed (O_NO_3_^–^_–H_EA^+^_). Besides interacting with the nitrate anions, in all the investigated mixtures, EA^+^ also forms hydrogen bonds with the MeOH molecules, and the H_EA^+^_–O_MeOH_*N*_coord_ decreases when more and more EAN is added to the sample, as a consequence of the significant increase of the IL anion–cation interactions. As concerns the interaction between the two kinds of cations present in our systems, namely, La^3+^ and EA^+^, they do not directly interact with each other, as expected. Conversely, the EA^+^ cations are found in the second solvation shell of the La^3+^ cation, as a result of their interactions with both nitrate anions and MeOH molecules that are located in the La^3+^ first coordination shell. Moreover, the number of EA^+^ cations belonging to the La^3+^ second coordination shell increases when more and more EA^+^ cations are added to the mixtures. Altogether, these results show that in the investigated systems a variety of different interactions exists and the system packs in a manner to maximize its interactions between the different moieties. Therefore, a complex network of interactions is formed resulting from the delicate balance among all of the different forces into play.

## Discussion and Conclusions

In this work, we have studied the structural properties of the La(NO_3_)_3_ salt dissolved into several mixtures of EAN and MeOH with χ_EAN_ ranging from 0 to 1. The first important result we obtained from both our MD simulations and EXAFS experimental data is that going from low-to-high χ_EAN_ values, the La^3+^ first solvation shell progressively loses MeOH molecules to accommodate more and more nitrate ligands. Very interestingly, with the exception of the pure EAN system where no MeOH molecules are present, the La^3+^ ion coordinates both MeOH molecules and nitrate anions in all of the investigated mixtures. On the one hand, the nitrate anion shows a very high affinity toward the La^3+^ cations which enable to bind to this cation also when few nitrate anions are present in the solution, namely, in the pure MeOH solvent. This is at variance with the behavior of La(NO_3_)_3_ in water where the salt is fully dissociated,^67^ as a consequence of the stronger solvation ability of water as compared to MeOH. On the other hand, even when the nitrate anions are present in great excess (such as in the mixture with χ_EAN_ = 0.8), they never saturate the La^3+^ first solvation shell, which still contains MeOH molecules. Even if the ratio of nitrate anions and MeOH molecules coordinated to La^3+^ strongly depends on the IL content of the mixture, the variation of the total first-shell coordination number (La–O_NO_3_^–^_ + La–O_MeOH_) is of two units going from χ_EAN_ = 0.0 to χ_EAN_ = 1.0: the La^3+^ ion prefers to form a 10-coordinated first-shell complex with a bicapped square antiprism geometry in pure MeOH and a 12-fold icosahedral structure in pure EAN. Moreover, a different behavior of the nitrate ligands is observed by increasing the IL content of the mixture: in pure MeOH, the nitrate anions enter the La^3+^ solvation shell by acting as monodentate ligands, while in the MeOH/EAN mixtures, the nitrate anions always prefer to bind La^3+^ in a monodentate way, but with a small percentage of bidentate coordination which becomes more and more significant with increasing EAN concentration. The orientation of the nitrate ligand in the coordination complex is also different when adopting the two different coordination modes: in the monodentate case, its orientation is very disordered and possesses a high orientational freedom, while a more ordered arrangement is formed when the nitrate ion acts as a bidentate ligand.

The MD findings are well corroborated by the EXAFS experimental data. In particular, the FT peak at about 3.8 Å is associated with both the linear La–O–N and La–N–O three-body configurations of the monodentate and bidentate ligands, respectively.^27,69^ The increase of the bidentate ligand number with increasing EAN content is thus confirmed by the higher intensity of this FT peak for the mixtures with a higher EAN concentration. Moreover, it is worth mentioning that the intensity of the FT first peak of the pure MeOH sample is slightly higher than those of the EAN mixtures despite the lower coordination number of the former sample. This trend could be due to a slight antiphase effect between the La–O sub-shells associated with the MeOH and nitrate ligands.

Altogether, our findings show that the MeOH molecules and the nitrate anions are able to compete with each other to bind the La^3+^ metal ion in the entire concentration range, resulting in a solvation complex which gradually evolves. This is a very interesting behavior which is at variance with the results obtained for other Ln^3+^ ions in IL mixtures with water. Independently, on the strong (such as Cl^–^) or weak (such as PF_6_^–^, Tf_2_N^–^ or TfO^–^) coordinating ability of the IL anions, a variation in the composition of the Ln^3+^ first solvation shell was found only in a narrow concentration range of the mixtures, with either only water or only anions coordinating the lanthanide ions outside such range.^70−73^ Conversely, here in all of the mixtures, the La^3+^ solvation complexes formed are composed of both MeOH molecules and nitrate anions, indicating that neither of the two ligands dominate over the other one. The same behavior has been recently observed for the La(Tf_2_N)_3_ salt dissolved into mixtures of acetonitrile and the 1,8-bis(3-methylimidazolium-1-yl)octane bistriflimide (C_8_(mim)_2_(Tf_2_N)_2_) IL: major changes were shown to take place in the La^3+^ first solvation shell when moving from pure acetonitrile to the pure IL, with a ratio between the acetonitrile and Tf_2_N^–^ ligands which strongly varies in the entire composition range.^60^ As compared to the latter system, one can expect that the presence of nitrate anions in the EAN/MeOH mixtures studied here, which have a stronger coordination ability as compared to Tf_2_N^–^, could result in a coordination shell formed only by nitrate anions in the mixtures with high EAN content. On the contrary, our results show that MeOH is able to compete with nitrate even when nitrate is present in great excess. This peculiar finding can be explained by the fact that forming such mixed structures, the system packs in a manner to maximize its interactions between all of the different moieties, namely, nitrate, ethylammonium, La^3+^, and MeOH, since all of these interactions have an important role in determining the overall structural arrangements formed in solution.

The overall geometries of the La^3+^ solvation complex and the spatial arrangement of the single first-shell ligands derive from the delicate balance between the maximization of electrostatic forces, the minimization of the repulsion among the ligands, and the maximization of the interactions with the IL cations belonging to the La^3+^ second solvation shell. Our findings thus suggest that the La^3+^ solvation structure formed in MeOH/EAN mixtures is able to adapt to changes in the composition, allowing the systems to reach the ideal compromise among all of the different forces into play. These results can help in the rationalization of the Ln^3+^ coordination chemistry in IL media, which is a key step to design IL systems for specific applications involving lanthanides.
